# Development of a Method for the Determination of Rifaximin and Rifampicin Residues in Foods of Animal Origin

**DOI:** 10.3390/molecules29194599

**Published:** 2024-09-27

**Authors:** Li-Ping Fan, Qi Tao, Xiao-Qiao Wang, Xiao-Hui Xu, Ya-Jun Yang, Jian-Yong Li

**Affiliations:** 1 Key Lab of New Animal Drug of Gansu Province, Key Lab of Veterinary Pharmaceutical Development of Ministry of Agriculture and Rural Affairs, Lanzhou Institute of Husbandry and Pharmaceutical Sciences of CAAS, Lanzhou 730050, China; 2 Key Laboratory of Pesticides and Veterinary Drugs Monitoring for State Market Regulation, Lanzhou Institute for Food and Drug Control, Lanzhou 730050, China

**Keywords:** rifaximin, rifampicin, drug residues, animal-origin foods, detection method

## Abstract

Rifaximin and rifampicin are good broad-spectrum antimicrobials. The irrational use of antimicrobial drugs in veterinary clinics could threaten public health and food safety. It is necessary to develop a reliable detection method of the residue for enhancing the rational supervision of the use of such drugs, reducing and slowing down the generation of bacterial resistance, and promoting animal food safety and human health. So, this study developed an LC-MS/MS method for the detection of rifaximin and rifampicin residues in animal-origin foods. The residual rifaximin and rifampicin of homogenized test materials were extracted with acetonitrile-dichloromethane solution or acetonitrile in the presence of anhydrous sodium sulfate and vitamin C, purified by dispersible solid phase extraction, determined by LC-MS/MS, and quantified by the internal standard method. The specificity, sensitivity, matrix effect, accuracy, and precision of the method were investigated in the edible tissues of cattle, swine, or chicken. In addition, the stability of the standard stock solution and the standard working solution was also investigated. The method was suitable for the muscle, liver, kidney, fat, milk, and eggs of cattle, swine, or chicken, as well as fish and shrimp. The specificity of the method was good, and the detection of the analytes was not affected by different matrices. Both the LOD and LOQ of the two analytes were 5 μg/kg and 10 μg/kg, respectively. The results of matrix effects in each tissue were in the range of 80–120%; there were no significant matrix effects. The average accuracy of rifaximin and rifampicin in different foodstuffs of animal origin was between 80% and 120%, and the method precision was below 20% (RSD). The proposed method showed good performance for determination, which could be employed for the extraction, purification, and detection of residual rifaximin and rifampicin in edible animal tissues. The pretreatment procedure of tissue samples was simple and feasible. The method was highly specific, stable, reliable, and with high sensitivity, accuracy, and precision, which met the requirements of quantitative detection of veterinary drug residues.

## 1. Introduction

**Figure 1:**
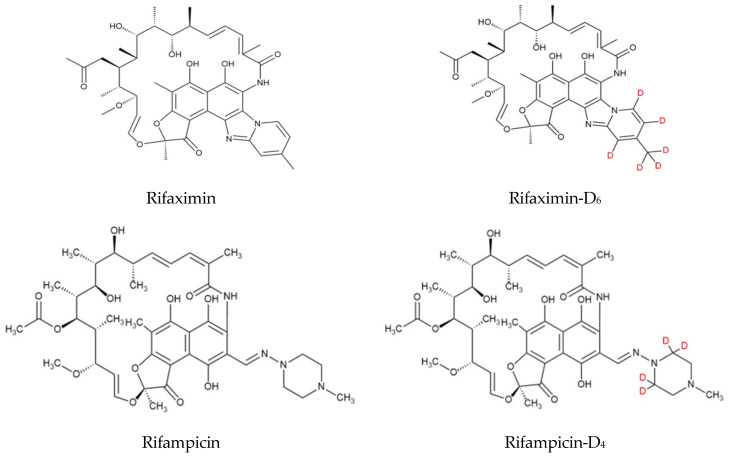
Structural formula of each compound.

Rifaximin and rifampicin are semi-synthetic rifamycin antibiotics. The structural formula is shown in Figure 1. Rifaximin has good antibacterial activity against Gram-positive bacteria such as *Staphylococcus aureus*, *Streptococcus*, and *Corynebacterium* and Gram-negative bacteria such as *Escherichia coli*, *Pasteurella*, and *Proteus*. Rifaximin intramammary infusion and intrauterine infusion have been authorized in China for the treatment of mastitis and endometritis in lactating dairy cows, as well as the prevention of mastitis in dry dairy cows [1]. Rifampicin has a strong antibacterial effect on *Mycobacterium tuberculosis* and also has a curative effect on Gram-positive or negative bacteria. It is mainly used to treat tuberculosis, meningitis, and *Staphylococcus aureus* infection, and can be used to treat trachoma. Rifampin can be used to control bacterial diseases of fish in aquaculture, but rifampicin has not yet been approved for veterinary clinical use.

In 2005, the Ministry of Agriculture Announcement No.560 [2] made it clear that rifamycin and its salts and esters are the latest antibacterial drugs used in medical clinical control and prohibited the use of rifamycins and their salts in veterinary clinics. If they are used in food animals and will cause drug resistance problems, they affect animal disease control, food safety, and human health. However, these two drugs have good antibacterial activity and may be used outside the prescribed indications in veterinary clinical practice or be used illegally. It has been reported that pathogens resistant to rifampicin have been found in aquaculture environments, aquatic animals, and swine [3,4,5]. Yunyu Tang et al. have reported that rifampicin has been detected in aquatic products [6].

Residues of antibiotics in foodstuffs of animal origin threaten food safety and public health. Effective monitoring of drug residues is an important measure of drug residue prevention and control. At present, only the entry–exit industry detection standards SN/T 2224-2008 [7] and China national standards GB 31659.5-2022 [8] can be used to detect rifaximin in swine muscle, liver, kidney, or milk, while there is no corresponding detection standard for rifaximin in other types of animal origin foods. In addition, there is also no standard method for the detection of rifampicin in animal-origin foods. To prevent these drugs from abuse, it is necessary to formulate corresponding detection methods for effectively monitoring the residues of rifaximin and rifampicin in animal-origin foods simultaneously, to ensure food safety.

Mass spectrometry is suitable for the analysis of molecules that have large molecular weights, complex chemical structures, and specific functional groups. Rifaximin and rifampicin are macrocyclic lactam compounds. These two molecules are easy to ionize and obtain high molecular ion peaks in mass spectrometry attributed to polar groups such as phenolic hydroxyl and amino groups and conjugated systems such as aromatic rings and conjugated double bonds. These can also form unique fragmental ion peaks in mass spectrometry due to heteroatoms and functional groups.

The liquid chromatography-tandem mass spectrometry (LC-MS/MS) method has been used to detect rifaximin in swine edible tissues and milk [7,8,9,10], as well as rifampin in aquatic products [11,12,13]. Multi-ion reaction monitoring (MRM) of LC-MS/MS has the characteristics of fast, high sensitivity, and high specificity, and is not interfered with by chemical noise. LC-MS/MS is the preferred method for the determination of rifampicin and rifaximin in animal-origin foods. The stable isotope-labeled internal standard has beyond-comparison advantages in LC-MS/MS detection. Therefore, the LC-MS/MS with isotope internal standard method was employed for the detection of rifampicin and rifaximin in animal-origin foods.

There are no regulations on the residue limits of rifaximin and rifampicin in animal foods in China, the US, and Japan. The EU authorizes rifaximin as limited to local treatment; its residual marker in milk is rifaximin and the maximum residue limit (MRL) in milk is 60 μg/kg [14]. There are no regulations on residue limits of rifampicin and rifaximin in other animal tissues. In China, only SN/T 2224-2008 was employed for the confirmation and detection of rifaximin residues in swine muscle, liver, kidney, or milk. There is no uniform standard method for rifampicin residue detection. The published papers mainly focused on the detection of aquatic product residues of rifampicin. At present, there is no simultaneous detection method for rifampicin and rifaximin residues in animal foods. Rifaximin and rifampicin have good antibacterial activity, so the possibility of illegal use is not ruled out. It is necessary to establish detection methods for rifaximin and rifampicin residues in animal foods and formulate corresponding standards to monitor the related residues in animal foods. This study aims to develop an LC-MS/MS method for the detection of rifaximin and rifampicin residues in animal foods in which the pretreatment procedure was simple and time-saving and could meet the requirement of the simultaneous detection of rifaximin and rifampicin in more edible tissues such as muscle, liver, kidney, fat, etc., in cattle, chicken, pig, fish, shrimp, etc.

## 2. Results

### 2.1. Optimization of Chromatographic Conditions and Mass Spectrum Conditions

The mobile phase elution gradient was optimized. The optimized gradient program is shown in Table 1.

The spectra of the precursor ion and product ion of each analyte and internal standard are shown in Figure S1. The MRM parameters of each compound were optimized in the SIM and MS/MS modes. The ions 786.3 > 754.2 and 823.3 > 791.0 were selected as the quantitative ions of rifaximin and rifampicin, respectively. The optimized MRM parameters are shown in Table 2.

### 2.2. Optimization of Extraction Procedure

When the 15 mL extraction solution was evaporated in a vacuum at 40 °C for a long time and redissolved, the spiked analytes in the samples could not be detected well. As rifaximin and rifampicin are polyphenolic hydroxyl compounds (see Figure 1), it is speculated that the analytes were oxidized during the process of long-term evaporation in a vacuum. Xin Yu [15] mentioned that the use of vitamin C (VC) and mercaptoethanol can prevent the oxidation of rifampicin from rifampicin-quinoid.

Because of VC adding and the extraction solution concentrated method improving, the oxidation of the analytes during processing has been inhibited. The results in Table 3 show that there was no significant difference in the supplemental amount of VC (20 mg, 50 mg, or 70 mg).

To investigate the influence of different concentration methods on the detection, the extract was concentrated to dryness at 40 °C by evaporation in a vacuum and nitrogen in water bath, respectively. The results showed that it took 30 h to concentrate a batch of samples (56 samples) by evaporation in a vacuum for only 1.5 h by nitrogen in a water bath. Meanwhile, rifampicin could not be detected and rifaximin was undetectable at low concentrations in the former; the response of rifaximin and rifampicin was good at LOD and LOQ in the latter.

The treatment effects of different extracts are shown in Table 4. In the experiment, it was found that the effect of acetonitrile-dichloromethane on the protein precipitation of milk and eggs was not as good as that of acetonitrile. After centrifugation, in the process of extracting the analyte in fat, the supernatant layer is under the fat layer, and it is not convenient to transfer the supernatant. Therefore, acetonitrile was used as the extraction solvent when milk, eggs, and fat were processed. Fat is solidified at room temperature and cannot be fully extracted, so when fat samples are processed, a 50 °C water bath is used to melt the fat to fully extract it. In addition, the tissue viscosity of shrimp is relatively high, and it cannot be dispersed when vortexed directly with extraction solvent. The dispersion effect of adding ceramic homogenizers was still not ideal. Before the treatment, 2 mL of water was added to the tube, and then the extraction solvent was added; the dispersion effect was ideal.

### 2.3. Method Validation

#### 2.3.1. Specificity

**Figure 2:**
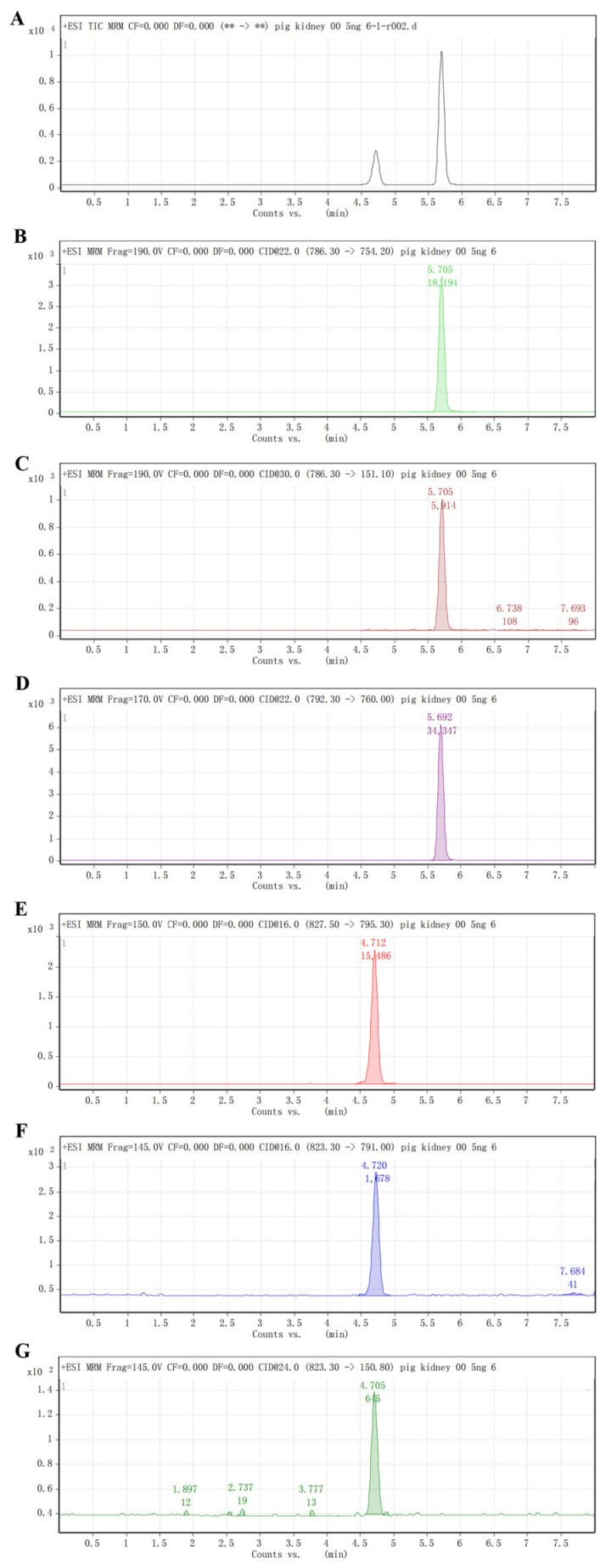
MRM chromatogram of sample (5 μg/kg) (**A**) TIC. (**B**) rifaximin 786.3 > 754.2. (**C**) rifaximin 786.3 > 151.1. (**D**) rifximin-D6 792.3 > 760.0. (**E**) rifampicin-D4 827.5 > 795.3. (**F**) rifampicin 823.3 > 791.0. (**G**) rifampicin 823.3 > 150.8.

The typical chromatograms are shown in Figure 2. The retention time of rifampicin and rifaximin was 3.4 and 3.9 min, respectively. The results showed that different blank matrices did not interfere with the detection of the analytes (Figure 2 and Figures S2–S4).

#### 2.3.2. Sensitivity

The LOD and LOQ were investigated by the detection results of analytes with low concentrations in different matrices. The results showed that the matrix of different edible tissues could not affect the detection of low concentrations of analytes. The SNR results are shown in Tables S1–S4. At 5 μg/kg and 10 μg/kg, the SNRs of the quantitative ion were greater than 3 and 10, respectively. The accuracy and precision results of low concentration in different matrices are in the range of 80–120% (RSD < 15%), as shown in Tables S5, S6, S11 and S12. Combined with the results of SNR, accuracy, and precision, LOD was 5 μg/kg and LOQ was 10 μg/kg for this method.

The results showed the CCα of rifaximin and rifampicin were 0.5 μg/kg and 1 μg/kg, respectively. This concentration is lower than the detection limit of the instrument, and cannot be calculated accurately. Therefore, the detection limit is still determined by low-concentration SNR and response.

#### 2.3.3. Matrix Effects

The matrix effect was investigated in tissues such as muscle, liver, kidney, fat, milk, and eggs of cattle, swine, or chicken, as well as fish and shrimp, and the results are shown in Table 5 and Table 6. The results of each tissue were in the range of 80–120%; there were no significant matrix effects.

#### 2.3.4. Linearity Range

The results showed that the linearity of each analyte was good in the range of 10 μg/kg to 300 μg/kg (Table 7).

#### 2.3.5. Accuracy and Precision

The accuracy and precision of the muscle, fat, kidney, and liver of cattle, swine, and chicken and other representative tissues such as milk, egg, fish, and shrimp were investigated. The results are shown in Tables S5–S16. The results showed that the accuracy and precision results met the requirements of quantitative detection (80–120%) when rifaximin-D_6_ was used as the internal standard for rifaximin and rifampicin-D_4_ was used as the internal standard for rifampicin.

#### 2.3.6. Stability of the Stock Solution

The results are shown in Table 8 and Table 9. The results showed that the standard stock solution of 1 mg/mL and 100 μg/mL mixed standard stock solution was stable under the condition of −20 °C for 2 months; the mixed standard working solution of 36 μg/mL and 2 μg/mL were stable under −20 °C for 1 month.

## 3. Discussion

Antimicrobial drugs are naturally occurring, semi-synthetic, or synthetic compounds with antimicrobial activity, which have shown tremendously good efficacy for use in food animals to improve the quality of life of the animals as well as to promote animal growth and feed efficiency [16,17,18,19]. Currently, approximately 80% of food animals are given drugs for part or even most of their life [20]. Drug residues exceeding the limits were most likely due to non-observance of withdrawal periods and over-labeling that could lead to contamination of food of animal origin [21,22]. Multiple veterinary drug residues are widely present in food of animal origin, posing a health hazard to consumers [23]. Antibacterial drug residues in milk may cause drug hypersensitivity reactions in milk consumers, manifesting as skin reactions, asthma, or other anaphylaxis [24,25]. Antimicrobials also disrupt the production of dairy products, reduce the acid and flavor produced during butter production, reduce milk curdling, and cause cheese to ripen poorly [26,27,28]. For these reasons, many countries and organizations, including the European Union, the United States of America, China, Japan, Canada, and New Zealand, have established MRL for veterinary drugs in foods of animal origin. Therefore, screening and detection of antimicrobial drug residues through validated methods in foods of animal origin is of great significance to ensure food safety.

Many methods of rifaximin detection have been reported, such as electrochemical and optical protocols for the detection and removal of an antibiotic drug rifaximin from wastewater [29], HPLC–PDA and HPLC for detection of rifaximin in plasma [30], and UPLC-MS/MS for detection of rifaximin residue in milk [31]. In addition to the usual method for rifampin detection, there are some new methods were reported, such as the synergistic activity of copper and molybdenum-based organic frameworks for the exceptional electrochemical detection of rifampicin in commercial dairy products [12] and concentration-regulated multi-color fluorescent carbon dots for the detection of rifampicin [32]. The method used in China to detect rifaximin residues is HPLC-MS/MS in SN/T 2224-2008 [7] and GB 31659.5-2022 [8]. HPLC-MS/MS was a common residue detection method and a method that can be implemented by almost all testing organizations.

The sample-processed method of the SN/T 2224-2008 is as follows: the sample was extracted twice with acetonitrile, the supernatant was concentrated to dry by rotary evaporation, and then redissolved the residue with 3 mL acetonitrile and purified by Waters oasis HLB solid phase extraction column [7]. The eluent was second-concentrated to dry and redissolved. Only one sample can be made at a time. The method took a long time due to two concentration processes. So, the optimized sample-processed procedure occurs after reference to the mentioned literature method. A method was developed such that the extracted solution was concentrated to dry just one time, and multiple samples could be concentrated at the same time. Compared with the standard SN/T 2224-2008, the proposed method in this research is simple and time-saving.

In addition, in GB 31659.5-2022, milk samples were extracted once with acetonitrile and purified by a hydrophilic–lipophilic solid phase extraction column. About 10 mL of the purified solution was concentrated by nitrogen blowing at 50 °C and redissolved [8]. The matrix-matched standard solution was quantified by the external standard method. The method of GB 31659.5-2022 was simple, easy to operate, and fast. However, the external standard method is easily affected by instrument stability and operation differences. There may be differences in the extraction efficiency of non-isotope internal standards, which makes the method inaccurate. The isotope internal standard method can reduce the error caused by the difference in instrument state and extraction efficiency and eliminate the matrix effect, too. Therefore, the stable isotope internal standard was selected in this method.

In addition, SN/T 2224-2008 and GB 31659.5-2022 can only be applied to the detection and confirmation of rifaximin in swine muscle, liver, kidney, or milk [7,8]. This method was used to investigate the edible tissues of cattle, swine, chickens, fish, and shrimp that may have food safety risks of rifaximin and rifampin, which can be applied to a wider range of food safety control.

## 4. Method and Materials

### 4.1. Reagent and Apparatus

Rifaximin (CAS: 80621-81-4), purity 95.7%, National Institute for Drug and Biological Products Control (Beijing, China), Lot: 130542-200601. Rifampicin (CAS: 13292-46-1), purity 98.8%, China Institute for Food and Drug Control (Beijing, China), Lot: 130496-201403). Rifaximin-D_6_, Toronto Research Chemicals INC (Toronto, ON, Canada), Lot: 3-YSW-91-6 (1.01 mg). Rifampicin-D_4_, TLC Pharmaceutical Standards Ltd. (Newmarket, ON, Canada), Lot: 1182-093A3 (1 mg).

Acetonitrile, mass spectrometric pure, Merck (Darmstadt, Germany). Formic acid, Mass spectrometric pure, Tokyo Chemical Co., Ltd., (Tokyo, Japan). Acetonitrile and Dichloromethane, analytical Reagent, Sinopharm Group Chemical Reagents Co., Ltd., (Shijiazhuang China). Vitamin C, analytical Reagent, Solarbio (Beijing China). Anhydrous Sodium sulfate, Sinopharm Group Chemical Reagents Co., Ltd., (Shijiazhuang China). Primary secondary amine (PSA) and Octadecylsilane (C_18_), Agilent Technologies (Santa Clara, CA, USA). 

Multifuge X3R high-capacity cryogenic centrifuge, Thermo Scientific, Waltham, MA, USA. Vortex Genie 2 Vortex Mixer, Scientific Industries, Bohemia, NY, USA. Mixer-400 tissue homogenizer, Buchi, Switzerland. AutoEVA-60 Automatic Parallel concentrator, Ruike Instrument (Xiamen, China) Co., Ltd. KQ-600DE type numerical control ultrasonic cleaner, working frequency 40 KHz, Kunshan Ultrasonic Instrument Equipment Co., Ltd. HH-S4 Thermostatic Water Bath, Zhengzhou Great Wall Technology Industry and Trade Co., Ltd.

LC-MS/MS with electrospray ionization (ESI) source are Agilent 1200-6410A and Agilent 1260-6460A, data acquisition and processing softwares are MassHunter B.01.04 (Agilent Technologies, USA).

### 4.2. Solution Preparation

#### 4.2.1. Standard Solution

Standard stock solution (1 mg/mL) with 10.0 mg of rifaximin and rifampicin were taken, precisely weighed, placed in a 10 mL volumetric flask, dissolved with mass spectral purity acetonitrile, and diluted to the scale, misce bene, stored at −20 °C. A mixed standard stock solution (100 µg/mL): 1 mL rifaximin and rifampicin standard stock solution with a mass concentration of 1 mg/mL was accurately measured in one 10 mL volumetric flask, diluted to the scale with mass spectral purity acetonitrile, misce bene, and stored at −20 °C. Serial mixed standard working solutions: A proper amount of mixed standard stock solution was accurately removed and diluted with mass spectral purity acetonitrile to prepare a serial mixed standard working solution with concentrations of 1, 2, 4, 6, 12, 36, and 60 μg/mL. The mixed standard working solutions were stored at −20 °C for a valid period of 1 month.

Internal standard stock solution (100 μg/mL), rifaximin-D_6_, and rifampicin-D_4_ were dissolved by mass spectral purity acetonitrile, mixed, and diluted to the scale, respectively, and stored at −20 °C. A mixed internal standard working solution (10 μg/mL): 1 mL rifaximin and 1 mL rifampicin internal standard stock solution was taken into one 10 mL volumetric flask and diluted with mass spectral purity acetonitrile to the scale, misce bene. The mixed internal standard working solution (4 μg/mL): 4 mL mixed internal standard was taken into a 10 mL volumetric flask, diluted with mass spectral purity acetonitrile to the scale, *misce bene*, and stored at −20 °C.

#### 4.2.2. Other Solution

Acetonitrile-dichloromethane, ACN-DCM (6 + 4): 200 mL dichloromethane and 300 mL acetonitrile (analytical pure) were mixed. In total, 0.01% formic acid solution: 100 μL formic acid was taken into a 1000 mL volumetric flask and diluted with water to scale. A 0.01% acetonitrile formate solution: 100 μL formic acid was taken into a 1000 mL volumetric flask and diluted with water to scale.

### 4.3. Sample Preparation

The edible tissues of swine, cattle, and chicken were homogenized. Fresh or thawed milk was homogenized. Eggshell was washed and removed, then egg whites and yolks were mixed and homogenized [33]. The head, bones, and internal organs of the fish were thrown away, and the edible parts such as muscle and skin were homogenized [34]. The head, shell, and intestinal glands of shrimp were removed away, and the residue was homogenized [35].

Accurately weigh 2.0 ± 0.02 g of the sample into a 50 mL centrifuge tube, then the mixed internal standard working solutions was added into. 10 mL ACN-DCM (6 + 4) (ACN for milk, eggs and fat) extraction solution were added into tubes, and mixed with vortex mixing for 1 min. Then, 50 mg of vitamin C and 2.0 g of anhydrous sodium sulfate were added, followed by vortex mixing for 1 min. The mixture was extracted using an ultrasonic water bath at room temperature for 10 min and mixed with vortex mixing for 1 min. The system was centrifuged at 4500× *g* for 10 min at 4 °C. The supernatant was transferred into another centrifuge tube. The residue was re-extracted with a 5 mL extraction solution, then two parts of supernatants were combined and dried under nitrogen gas at 40 °C water baths. The residue was dissolved with 2 mL of ACN, then 50 mg PSA and 50 mg C_18_ were added. The mixture was vortex-mixed for 1 min and centrifuged. The supernatant was determined by LC-MS/MS after filtering through a syringe filter with a pore size of 0.22 μm.

### 4.4. HPLC–MS/MS Analysis

A mass spectrometer with an electrospray ionization source interface operated in the positive ion of MRM mode was used for LC-MS/MS analysis. The internal standard method was used for quantification by a calibration curve of standard solutions.

#### 4.4.1. Chromatographic Condition

Agilent Eclipse Plus C_18_ column, 3.0 mm × 100 mm, 1.8 μm. Mobile phase A was 0.01% formic acid acetonitrile and phase B was 0.01% formic acid. Column temperature 35 °C, injection volume 10 μL, injector temperature 4 °C. Before each needle injection, the needle was washed automatically for 10 s with the needle washing solution.

#### 4.4.2. Mass Spectrometer Conditions

Electrospray ion source (ESI) with positive ion mode. Capillary voltage + 4000 V. Nebulizer pressure 30 psi, drying gas (N_2_) temperature 350 °C and flow 10 L/min. The electron multiplier voltage increment (ΔEMV) was +600 V and +200 V for Agilent 6410A and 6460A, respectively. Quantitative detection was performed in the multi-ion reaction monitoring (MRM) mode.

### 4.5. Investigation of Method

The method was validated according to the guidelines for the verification of quantitative analysis methods of biological samples in general rule 9012 [36] of Pharmacopoeia of the People’s Republic of China (PRC) and the Guidelines for the elimination of veterinary drug residues [34] were announced by the ministry of agriculture and rural affairs of PRC No. 326. The calculation of the decision limits was guided by in the Decision 2002/657/EC [37].

#### 4.5.1. Investigation of Specificity

The specificity of the method was investigated by a mixed standard solution, blank matrix, and blank matrix with only one internal standard and sample (5 μg/kg).

#### 4.5.2. Investigation of Sensitivity

The signal-to-noise ratio (SNR) was calculated by the method of peak height vs. peak height, and the concentration with SNR ≥ 3 was taken as the limit of detection (LOD). Combined with the results of accuracy and precision tests, the concentration of signal-to-noise ratio SNR ≥ 10 was taken as the limit of quantitation (LOQ).

A calculation was performed for the decision limit (CC*α*) and the detection capability (CC*β*) for banned substances. A series of working samples with rifaximin and rifampicin contents of 3 μg/kg, 5 μg/kg, 7 μg/kg, 9 μg/kg, 11 μg/kg, 13 μg/kg, and 15 μg/kg were prepared with blank matrix and detected after pretreatment according to the method. With the peak area of quantitative ions as the vertical coordinate (y) and the concentration as the horizontal coordinate (x), the standard curve is drawn to find the regression equation, y = ax + b. Draw the standard curve in parallel 6 times. Find the mean value of b b ± SD, CCα = b + 2.33SD. Rifampicin and rifampicin standard solution were added into the blank matrix with the concentration of CCα, and 20 samples were prepared in parallel for each matrix. The content and average value of each compound were determined (C ± SD), CCβ = CCα + 1.64SD.

#### 4.5.3. Investigation of Matrix Effects

The matrix factors of each analyte and internal standard were calculated by calculating the ratio of the response of the analyte in the presence of the matrix to the corresponding response without the matrix. The matrix effect was calculated by internal standard normalization.

#### 4.5.4. Investigation of Linearity Range

The mixed standard solutions of rifaximin and rifampin were prepared with the concentrations of 5 ng/mL, 10 ng/mL, 20 ng/mL, 30 ng/mL, 60 ng/mL, 180 ng/mL, and 300 ng/mL, and the concentrations of rifaximin-D6 and rifampin-D4 were 20 ng/mL. The ratios of the quantitative ion responses of rifaximin and rifampicin to their internal standard were used as the ordinate, and the ratios of the concentrations of rifaximin and rifampicin to the concentrations of their internal standard were used as the abscissa. The appropriate weight was selected for linear regression, and the standard curves were drawn.

#### 4.5.5. Investigation of Accuracy and Precision

Different concentrations of blank addition test materials were prepared for different tissues. The addition concentrations of rifaximin and rifampin in different tissues were 5 μg/kg, 10 μg/kg, 20 μg/kg, 30 μg/kg, 60 μg/kg and 180 μg/kg. The addition concentrations of internal standard rifaximin-D6 and rifampin-D4 were both 20 μg/kg. Six samples were prepared at each concentration level. According to the established method of detection and quantitative calculation, the accuracy of the method and the intra-batch and inter-batch precision were investigated.

#### 4.5.6. Investigation of Stability of Standard Stock Solution

The standard stock solution with a concentration of approximately 1 mg/mL and 100 μg/mL mixed standard stock solution, as well as the concentration of 36 μg/mL and 2 μg/mL mixed standard solution, were stored at −20 °C for a certain period, properly diluted, and quantified with the newly prepared standard solution.

## 5. Conclusions

A method for the residue extraction, purification, and detection of rifaximin and rifampicin in animal edible tissues was established with LC-MS/MS. The method was suitable for the muscle, liver, kidney, fat, milk, and eggs of cattle, swine, or chicken, as well as fish and shrimp. The method is specific, stable, and reliable, and the pretreatment of tissue samples is simple and feasible, with high accuracy and sensitivity, which can meet the requirements of quantitative detection of veterinary drug residues. Compared with the present standard methods, this method takes less time and has simple pretreatment, which can meet the requirements of rapid qualitative and quantitative detection of large quantities of samples. At the same time, the scope of application is expanded to provide detection and monitoring in more animal edible tissues, to meet the simultaneous detection and monitoring of rifampicin and rifaximin, and to provide a reference for food safety monitoring, especially the risk control caused by the abuse or illegal application of rifamycins.

## Figures and Tables

**Table 1:** The gradient program for liquid chromatography.

Time (min)	A%	B%	Flow Rate (mL/min)
0	10	90	0.4
1.0	10	90	0.4
2.0	70	30	0.4
4.0	70	30	0.4
5.5	10	90	0.4
8.0	10	90	0.4

**Table 2:** MRM parameters and characteristic ions of each compound.

Compound	Prec Ion *m/z*	Prod Ion *m/z*	Fragmentor V	CE eV	Polarity
rifaximin	786.3	754.2 *	190	22	+
	786.3	151.1	190	30	+
rifaximin-D_6_	792.3	760.0 *	170	22	+
rifampicin	823.3	791.0 *	145	16	+
	823.3	150.8	145	24	+
rifampicin-D_4_	827.5	795.3 *	150	16	+

Note: *: Ions are quantitative ions. Prec Ion: Precursor Ion, Prod Ion: Product Ion CE: Collision voltage, *m*/*z*: mass-to-charge ratio, V/eV: Unit of voltage, Polarity: Mode of ionization, +: Positive mode.

**Table 3:** Effects of VC on the detection.

Dosage of VC	Method of Concentration	Recovery %	
		Rifaximin	Rifampicin
20 mg	dry with nitrogen in water bath	96.7	95.3
50 mg		101.2	95.7
70 mg		98.1	94.4

**Table 4:** Treatment effects of different extracts.

Tissue	Acetonitrile-Dichloromethane (6 + 4)			Acetonitrile			Methanol-Acetonitrile (3 + 7)		
	Dispersion	Protein Precipitation	Supernatant Extract Transfer	Dispersion	Protein Precipitation	Supernatant Extract Transfer	Dispersion	Protein Precipitation	Supernatant Extract Transfer
fat	√	√	×	√	√	√	√	√	×
egg	√	+	√	√	√	√	√	+	×
milk	√	+	√	√	√	√	√	+	×
shrimp	√	√	√	×	√	√	×	+	×
fish	√	√	√	+	√	√	√	+	×
muscle	√	√	√	+	√	√	√	+	×
liver	√	√	√	+	√	√	√	+	×
kidney	√	√	√	+	√	√	√	+	×

Note: √, Good, means good dispersion, good protein precipitation, and easy removal of the extract. +, Fair, means it can be dispersed but still has small clumps, protein can precipitate but not completely, there is residue in the supernatant or transfer, and precipitates are easily dispersed. ×, Poor, that is, cannot be dispersed, cannot precipitate protein, and there are more residues in the supernatant to affect the purification.

**Table 5:** Matrix effects of rifaximin in different tissues.

Animal	Tissue	Result %					
		LOD 5 μg/kg	LOQ 10 μg/kg	2LOQ 20 μg/kg	MRL 60 μg/kg	2MRL 120 μg/kg	3MRL 180 μg/kg
cattle	muscle	105.6	102.1	97.6	105.8	99.5	108.6
	fat	105.2	97.4	95.3	102.0	99.7	102.1
	liver	98.5	99.0	93.8	96.0	108.0	101.1
	kidney	94.9	97.1	97.4	104.0	97.1	98.6
	milk	96.9	103.1	97.0	95.8	100.1	93.7
swine	muscle	107.6	99.9	100.5	96.1	103.0	——
	kidney	101.5	90.8	97.6	104.0	92.6	92.8
	skin fat	103.0	——	102.2	92.3	96.2	91.4
chicken	muscle	101.2	104.7	98.3	102.7	100.7	117.6
	skin fat	102.8	98.0	98.6	100.0	102.0	92.6
	liver	100.6	96.1	98.5	102.2	98.4	99.3
	egg	99.4	101.9	106.0	109.2	106.5	99.1
fish	skin muscle	91.0	96.2	94.5	88.7	95.6	96.9
shrimp	muscle	99.2	98.3	101.4	101.7	102.4	100.4

**Table 6:** Matrix effects of rifampicin in different tissues.

Animal	Tissue	Result %					
		LOD 5 μg/kg	LOQ 10 μg/kg	2LOQ 20 μg/kg	MRL 60 μg/kg	2MRL 120 μg/kg	3MRL 180 μg/kg
cattle	muscle	118.3	111.1	104.1	111.9	94.9	101.6
	fat	115.7	107.3	101.0	108.4	104.1	101.8
	liver	98.3	104.7	95.2	98.3	106.4	102.6
	kidney	99.0	105.9	101.1	105.0	95.4	99.5
	milk	109.5	106.3	103.1	95.1	103.1	96.1
swine	muscle	118.8	——	109.5	127.1	93.4	98.8
	kidney	98.0	86.0	91.6	103.2	93.5	100.1
	skin fat	112.4	——	113.8	105.7	97.9	89.5
chicken	muscle	113.6	117.7	97.3	107.9	92.2	108.3
	skin fat	107.9	112.3	96.5	95.4	97.9	85.9
	liver	95.1	90.5	91.1	103.7	94.9	99.2
	egg	110.2	113.1	113.7	113.7	105.7	101.0
fish	skin muscle	104.0	98.3	99.7	87.6	98.1	89.0
shrimp	muscle	103.1	100.9	103.0	98.0	103.5	102.8

**Table 7:** Typical calibration curve equation of each analyte.

Compound	Linearity Range	Weight	Standard Curve Equation	*R* ^2^
rifaximin	10 μg/kg–300 μg/kg	1/*x*	*y* = 1.7726*x* + 0.0457	0.9960
			*y* = 1.9007*x* − 0.0667	0.9991
			*y* = 1.6562*x* + 0.0244	0.9991
			*y* = 2.0479*x* + 0.0343	0.9990
			*y* = 2.4088*x* − 0.2063	0.9991
			*y* = 1.7781*x* − 0.0112	0.9901
			*y* = 2.5775*x* − 0.2717	0.9986
rifampicin	10 μg/kg–300 μg/kg	1/*x*^2^	*y* = 0.7500*x* − 0.0338	0.9981
			*y* = 0.8112*x* − 0.0561	0.9965
			*y* = 0.6285*x* − 0.0642	0.9967
			*y* = 0.6683*x* − 0.0490	0.9941
			*y* = 0.4351*x* − 0.0250	0.9961
			*y* = 0.6330*x* − 0.0061	0.9937
			*y* = 0.4885*x* − 0.1870	0.9920

**Table 8:** The stability of the standard stock solution.

Concentration	Compound	1 Month %	2 Month %
1 mg/mL	rifaximin	102.5	93.8
	rifampicin	97.6	98.0
100 μg/mL	rifaximin	94.1	109.3
	rifampicin	94.0	97.6

**Table 9:** Stability of the mixed standard working solution.

Concentration	Compound	7 d %	21 d %	30 d %
2 μg/mL	rifaximin	98.6	101.5	100.1
	rifampicin	101.0	100.0	94.7
36 μg/mL	rifaximin	98.8	98.8	97.7
	rifampicin	100.6	97.7	93.8

## Data Availability

The data that support the findings of this study are available from the corresponding author upon reasonable request. Some data may not be made available because of privacy or ethical restrictions.

## Supplementary Materials

The following supporting information can be downloaded at: https://www.mdpi.com/article/10.3390/molecules29194599/s1, Figure S1: The scan chromatogram of each compound. A-Precursor ion scan of rifaximin, a-product ion scan of rifaximin. B-Precursor ion scan of rifaximin-D6, b-product ion scan of rifaximin-D6. C-Precursor ion scan of rifampicin, c-product ion scan of rifampicin. D-Precursor ion scan of rifampicin-D4, d-product ion scan of rifampicin-D4. Figure S2: TIC and MRM chromatogram of mixed standard solution. A. TIC B. Rifaximin 786.3 > 754.2*. C. Rifaximin-d6 792.3 > 760.0. D. Rifampicin-d4 827.5 > 795.3. E. Rifampicin 823.3 > 791.0*. Figure S3: Blank matrix MRM chromatogram. A. TIC. B. rifaximin 786.3 > 754.2*. C. rifaximin 786.3 > 151.1. D. rifximin-D6 792.3 > 760.0. E. rifampicin-D4 827.5 > 795.3, F. rifampicin 823.3 > 791.0*. G. rifampicin 823.3 > 150.8. Figure S4: Blank matrix with internal standard MRM chromatogram. A. TIC. B. rifaximin 786.3 > 754.2*. C. rifaximin 786.3 > 151.1. D. rifximin-D6 792.3 > 760.0. E. rifampicin-D4 827.5 > 795.3. F. rifampicin 823.3 > 791.0*. G. rifampicin 823.3 > 150.8. Table S1: SNR of LOD for rifaximin. Table S2: SNR of LOQ for rifaximin. Table S3: SNR of LOD for rifampicin. Table S4: SNR of LOQ for rifampicin. Table S5: Accuracy and precision of the method for rifaximin-LOD. Table S6: Accuracy and precision of the method for rifaximin-LOQ. Table S7: Accuracy and precision of the method for rifaximin-Low (20 ng/g). Table S8: Accuracy and precision of the method for rifaximin-Medium (30 ng/g). Table S9: Accuracy and precision of the method for rifaximin-MRL (60 ng/g). Table S10: Accuracy and precision of the method for rifaximin-High (180 ng/g). Table S11: Accuracy and precision of the method for rifampicin-LOD. Table S12: Accuracy and precision of the method for rifampicin-LOQ. Table S13: Accuracy and precision of the method for rifampicin-2LOQ. Table S14: Accuracy and precision of the method for rifampicin-Low (30 ng/g). Table S15: Accuracy and precision of the method for rifampicin-Medium (60 ng/g). Table S16: Accuracy and precision of the method for rifampicin-High (180 ng/g).
