# Supplementary material for: Development of a Method for the Determination of Rifaximin and Rifampicin Residues in Foods of Animal Origin
Source: Molecules. 2024 Sep 27;29(19):4599. doi: 10.3390/molecules29194599 (PMC11477573; doi:10.3390/molecules29194599)
Supplement: Supplementary file 1 [file molecules-29-04599-s001.zip › molecules-3105512-supplementary.pdf]

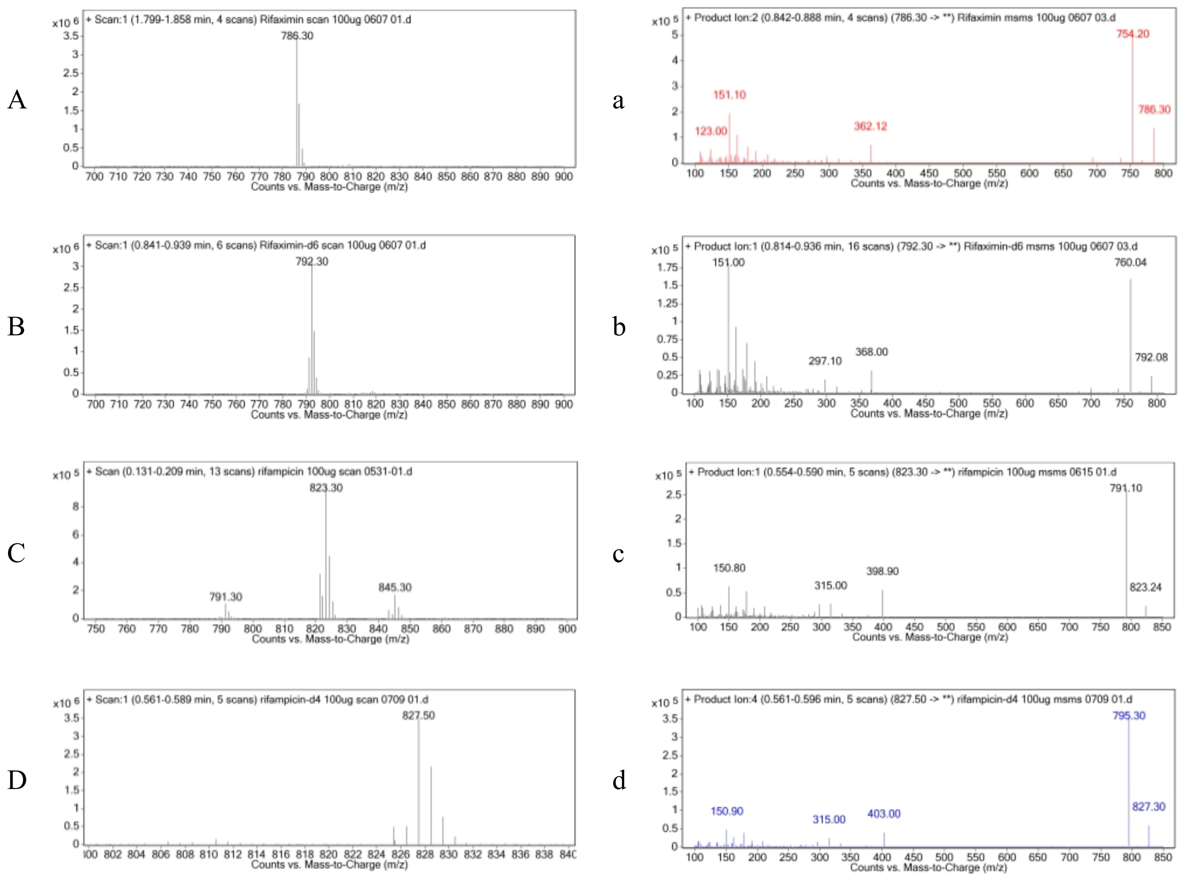

Figure S1 The scan chromatogram of each compound. **A**-Precursor ion scan of rifaximin, **a**-product ion scan of rifaximin. **B**-Precursor ion scan of rifaximin-D_6, **b**-product ion scan of rifaximin-D_6. **C**-Precursor ion scan of rifampicin, **c**-product ion scan of rifampicin. **D**-Precursor ion scan of rifampicin-D_4, **d**-product ion scan of rifampicin-D_4.

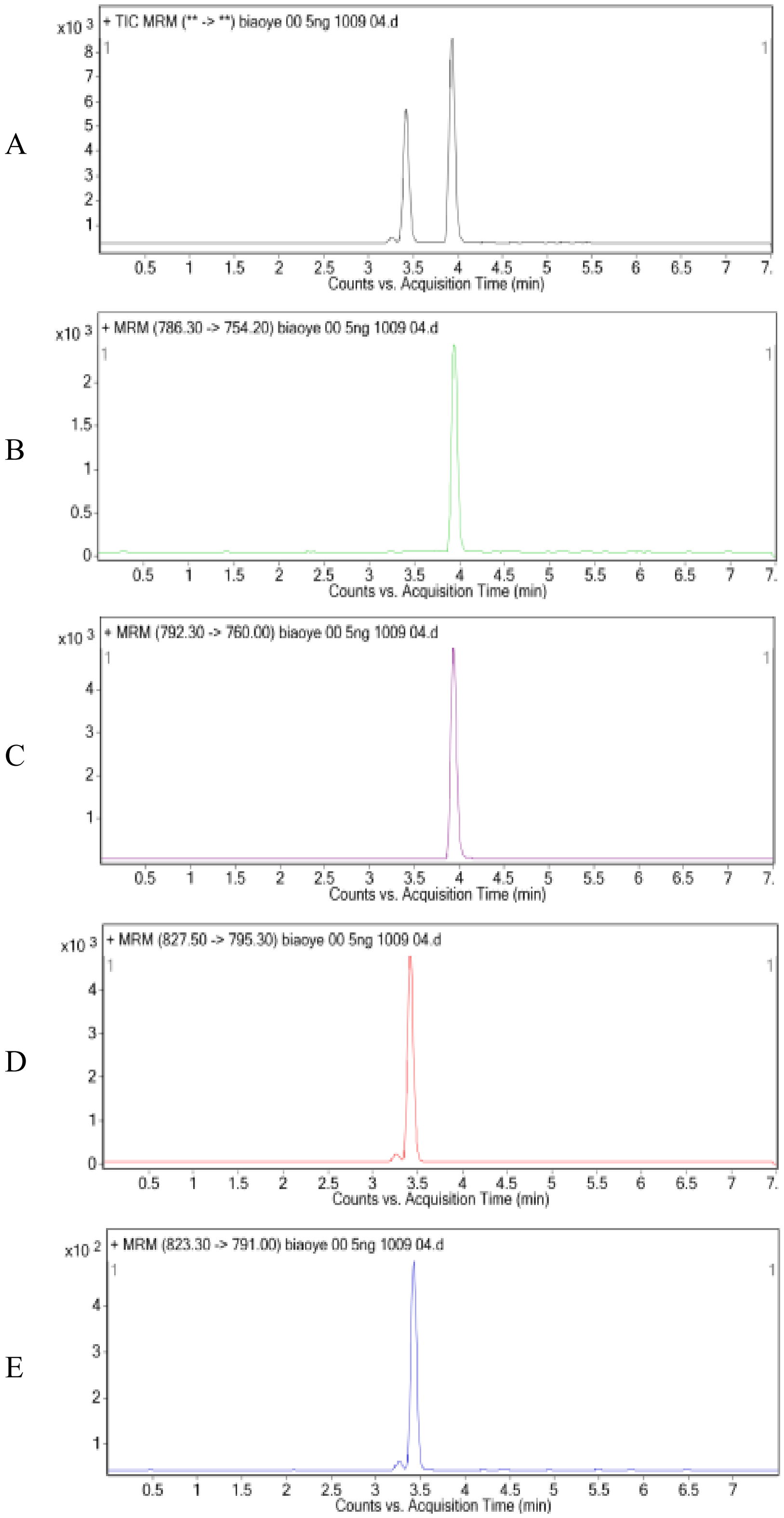

Figure S2 TIC and MRM chromatogram of mixed standard solution. A. TIC B. Rifaximin 786.3>754.2*. C. Rifaximin-d6 792.3>760.0. D. Rifampicin-d4 827.5>795.3. E. Rifampicin 823.3>791.0*.

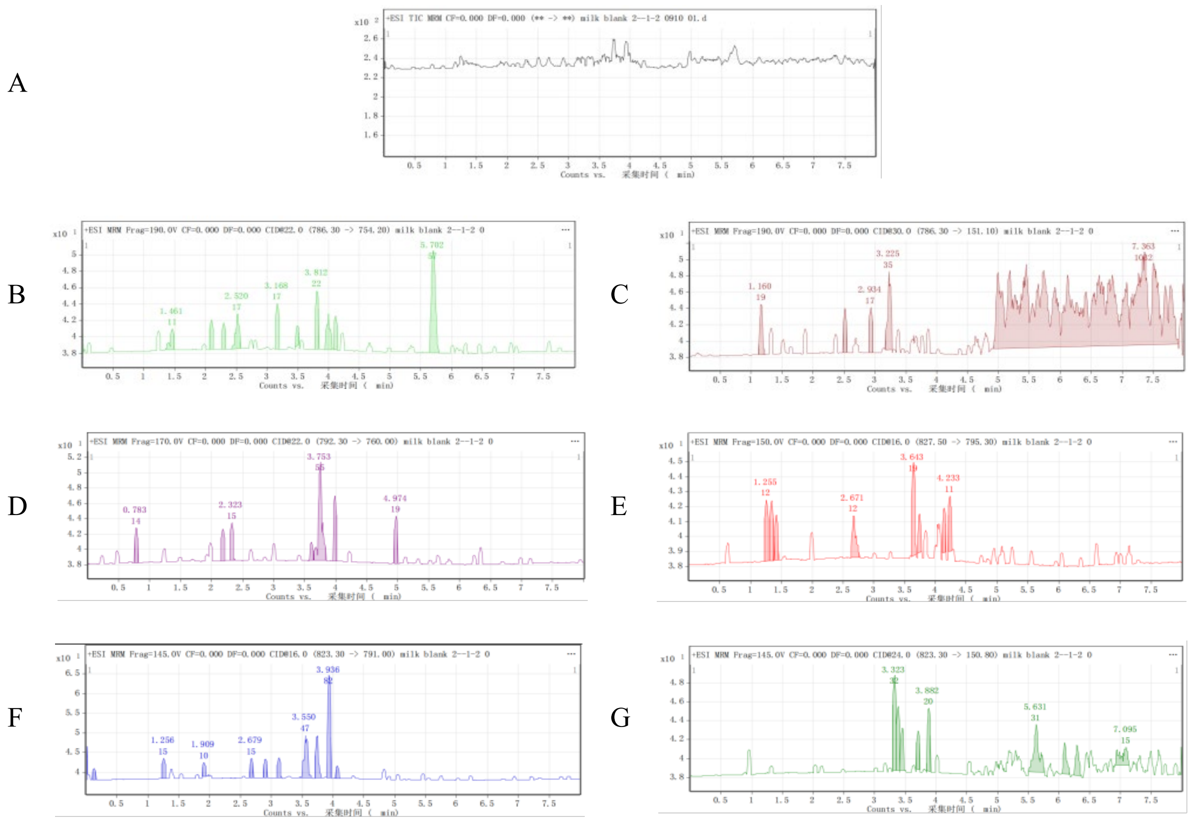

Figure S3 Blank matrix MRM chromatogram. A. TIC. B. rifaximin 786.3>754.2*. C. rifaximin 786.3>151.1. D. rifximin-D6 792.3>760.0. E. rifampicin-D4 827.5>795.3, F. rifampicin 823.3>791.0*. G. rifampicin 823.3>150.8.

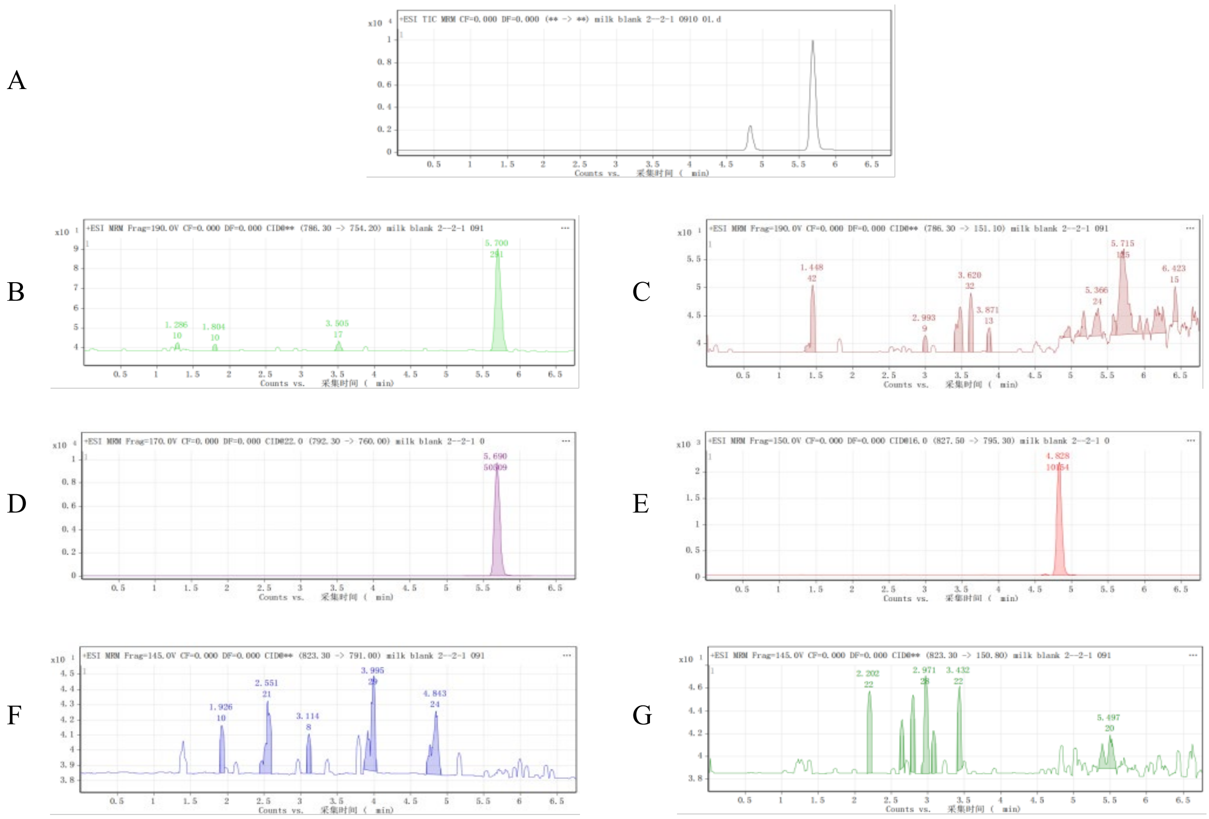

Figure S4 Blank matrix with internal standard MRM chromatogram. A. TIC. B. rifaximin 786.3>754.2*. C. rifaximin 786.3>151.1. D. rifximin-D6 792.3>760.0. E. rifampicin-D4 827.5>795.3. F. rifampicin 823.3>791.0*. G. rifampicin 823.3>150.8.

Table S1 SNR of LOD for rifaximin

animal	tissue	SNR (peak to peak)					
		**1**	**2**	**3**	**4**	**5**	**6**
cattle	muscle	237.4	479.3	385.9	146.3	171.3	172.3
	fat	49.9	45.5	41.6	112.7	48.8	118.0
	liver	105.1	87.8	122.8	107.8	140.1	155.7
	kidney	184.8	246.0	129.2	334.1	251.9	266.2
	milk	190.7	264.2	247.8	189.2	1119.6	654.5
swine	muscle	757.5	510.7	10448.0	infinity	6772.2	infinity
	skin+fat	431.0	400.6	1136.5	362.7	1689.5	313.0
	liver	1167.1	735.7	infinity	1434.3	7478.0	1440.7
	kidney	1285.5	993.2	953.1	1806.9	997.6	1880.7
chicken	muscle	673.1	2860.2	982.3	2102.4	774.5	972.8
	skin+fat	949.1	1179.4	433.7	515.1	784.1	914.6
	liver	387.9	518.8	799.5	741.7	959.4	1187.5
	egg	217.0	339.1	580.6	460.5	451.2	659.9
fish	skin+muscle	179.9	164.8	119.8	227.2	148.3	261.2
shrimp	muscle	315.0	244.7	305.1	467.9	525.4	579.4

Table S2 SNR of LOQ for rifaximin

animal	tissue	SNR (peak to peak)					
		**1**	**2**	**3**	**4**	**5**	**6**
cattle	muscle	938.3	734.1	840.5	555.9	1122.0	833.7
	fat	145.9	58.1	73.4	79.6	83.2	82.2
	liver	110.1	147.4	175.5	299.9	445.0	176.9
	kidney	279.6	246.0	129.2	350.4	152.7	322.2
	milk	610.7	425.2	248.0	453.6	423.4	580.3
swine	muscle	833.3	2203.1	1954.7	infinity	2488.5	1360.9
	skin+fat	636.7	568.9	2548.4	1265.2	1527.9	2874.5
	liver	1100.3	3156.1	2040.5	1163.9	1658.4	2666.8
	kidney	1300.0	1424.0	959.1	2052.8	2000.2	3150.4
chicken	muscle	1436.8	518.7	1985.7	1700.8	817.2	1905.4
	skin+fat	711.2	650.0	1012.1	861.4	1000.2	658.7
	liver	2744.9	2681.8	1979.6	1329.8	1038.1	1552.3
	egg	486.5	1697.0	682.3	967.1	725.1	674.6
fish	skin+muscle	285.0	182.2	286.6	226.7	163.8	248.9
shrimp	muscle	839.9	878.8	471.6	640.9	1030.3	502.9

Table S3 SNR of LOD for rifampicin

animal	tissue	SNR (peak to peak)					
		**1**	**2**	**3**	**4**	**5**	**6**
cattle	muscle	123.0	19.1	205.2	127.2	152.4	154.5
	fat	15.2	26.6	26.0	9.0	32.1	36.5
	liver	22.3	11.9	15.7	11.9	23.5	12.7
	kidney	23.7	45.9	30.8	32.3	30.5	25.5
	milk	200.4	199.9	32.5	19.7	254.3	38.9
swine	muscle	423.1	1034.2	329.6	1860.1	758.2	1053.5
	skin+fat	2265.4	1048.9	349.7	1307.1	341.8	1531.7
	liver	6943.3	508.4	5302.1	663.9	775.8	4793.9
	kidney	2588.9	100.8	1014.0	infinity	808.9	123.2
chicken	muscle	2721.6	200.9	210.2	463.5	3844.0	463.9
	skin+fat	209.4	128.6	1436.8	337.5	780.7	449.7
	liver	901.4	1953.1	infinity	6125.7	115.3	infinity
	egg	128.8	63.7	438.3	41.3	446.1	123.6
fish	skin+muscle	7.8	5.7	7.7	6.9	6.6	8.0
shrimp	muscle	253.7	507.8	188.6	614.4	559.2	147.0

Table S4 SNR of LOQ for rifampicin

animal	tissue	SNR (peak to peak)					
		1	2	3	4	5	6
cattle	muscle	269.5	197.0	411.9	131.4	47.9	228.4
	fat	82.4	46.3	71.6	77.2	40.9	102.3
	liver	54.9	55.2	48.8	37.1	45.0	41.0
	kidney	49.2	60.6	68.9	63.9	58.5	51.5
	milk	236.7	70.0	463.1	154.8	344.0	155.6
swine	muscle	135.8	106.7	250.4	153.3	2150.7	190.9
	skin+fat	978.2	1907.6	8418.8	503.3	276.1	613.0
	liver	1038.5	1831.0	655.0	infinity	3398.0	1271.3
	kidney	247.7	289.6	8776.3	12772.0	304.3	662.3
chicken	muscle	401.5	554.3	infinity	207.9	828.1	786.8
	skin+fat	infinity	1580.0	418.8	635.5	303.4	348.7
	liver	506.2	541.8	1837.3	infinity	1585.8	infinity
	egg	510.2	433.2	167.3	833.7	139.7	1022.6
fish	skin+muscle	9.2	17.4	21.2	5.8	14.7	16.4
shrimp	muscle	331.5	1021.6	1842.9	339.2	751.7	882.8

Table S5 Accuracy and precision of the method for rifaximin-LOD

animal	tissue	accuracy %		precision %	
		in-batch	between batch	in-batch	between batch
cattle	muscle	86.9-111.9	101.9	2.5-5.7	11.5
	fat	102.5-112.1	106.9	4.2-8.5	7.9
	liver	99.7-114.6	105.9	4.1-6.6	8.1
	kidney	92.4-108.7	102.2	3.7-4.2	8.0
	milk	97.1-107.9	100.8	4.0-4.9	6.6
swine	muscle	112.6	——	2.7	——
	skin+fat	101.4	——	2.7	——
	liver	97.6	——	2.5	——
	kidney	100.0	——	1.4	——
chicken	muscle	98.2	——	2.8	——
	skin+fat	102.6	——	3.4	——
	liver	108.1	——	6.2	——
	egg	97.8-109.1	105.3	1.5-11.0	8.4
fish	skin+muscle	101.3-114.0	107.4	3.2-4.9	6.3
shrimp	muscle	92.2-119.1	106.5	1.8-3.6	10.9

Table S6 Accuracy and precision of the method for rifaximin-LOQ

animal	tissue	accuracy %		precision %	
		in-batch	between batch	in-batch	between batch
cattle	muscle	87.4-102.2	95.6	1.1-7.0	7.9
	fat	97.2-105.6	102.0	3.0-9.9	7.8
	liver	97.7-102.0	99.6	2.5-4.9	4.2
	kidney	95.9-113.5	106.0	2.7-3.9	8.0
	milk	90.5-103.9	97.8	1.3-2.9	6.2
swine	muscle	95.5	——	4.1	——
	skin+fat	100.5	——	4.2	——
	liver	99.0	——	2.2	——
	kidney	103.1	——	2.0	——
chicken	muscle	101.9	——	2.8	——
	skin+fat	111.8	——	2.5	——
	liver	107.0	——	2.8	——
	egg	99.6	103.9	1.4-2.8	3.6
fish	skin+muscle	93.5-103.2	97.6	2.0-3.6	5.1
shrimp	muscle	99.2-107.2	104.1	2.1-3.0	4.2

Table S7 Accuracy and precision of the method for rifaximin-Low (20 ng/g)

animal	tissue	accuracy %		precision %	
		in-batch	between batch	in-batch	between batch
cattle	muscle	96.2-102.6	99.2	2.0-3.6	4.0
	fat	93.6-108.8	100.5	3.1-8.4	10.0
	liver	95.1-103.3	98.6	2.5-9.7	8.5
	kidney	98.0-100.4	98.8	3.2-5.2	4.0
	milk	94.5-106.6	101.3	2.8-4.4	6.2
swine	muscle	94.0	——	4.8	——
	skin+fat	99.0	——	5.6	——
	liver	92.9	——	3.0	——
	kidney	94.2	——	3.2	——
chicken	muscle	101.8	——	3.3	——
	skin+fat	103.0	——	4.2	——
	liver	97.5	——	4.8	——
	egg	98.3-100.4	99.3	1.7-6.5	4.2
fish	skin+muscle	88.9-96.9	92.3	2.0-7.0	5.6
shrimp	muscle	97.6-105.3	101.0	1.5-6.2	5.2

Table S8 Accuracy and precision of the method for rifaximin-Medium (30 ng/g)

animal	tissue	accuracy %		precision %	
		in-batch	between batch	in-batch	between batch
cattle	muscle	94.3-103.8	99.8	3.9-4.8	5.8
	fat	88.4-106.6	99.7	1.9-6.2	7.6
	liver	91.7-99.9	95.9	4.8-10.2	7.7
	kidney	99.1-116.4	108.6	2.1-4.7	7.5
	milk	95.7-110.0	104.2	3.6-3.9	7.1
swine	muscle	96.6	——	5.2	——
	skin+fat	99.3	——	3.6	——
	liver	93.7	——	2.3	——
	kidney	92.3	——	4.4	——
chicken	muscle	101.2	——	10.1	——
	skin+fat	105.1	——	9.8	——
	liver	95.4	——	3.0	——
	egg	96.7-104.2	100.0	1.8-2.9	3.8
fish	skin+muscle	89.8-99.2	95.1	1.0-1.9	4.5
shrimp	muscle	97.7-104.0	100.9	1.0-7.9	5.1

Table S9 Accuracy and precision of the method for rifaximin-MRL (60 ng/g)

**animal**	**tissue**	**accuracy %**		**precision %**	
		**in-batch**	**between batch**	**in-batch**	**between batch**
cattle	muscle	99.1-104.4	102.0	1.5-3.4	3.4
	fat	92.6-106.5	100.1	2.0-3.9	6.8
	liver	96.9-99.7	98.6	2.1-4.7	3.4
	kidney	99.7-102.5	101.3	1.7-8.4	5.0
	milk	98.5-115.5	104.8	2.3-5.1	8.3
swine	muscle	96.9	——	3.6	——
	skin+fat	97.7	——	7.2	——
	liver	96.0	——	2.1	——
	kidney	92.9	——	3.0	——
chicken	muscle	103.1	——	3.7	——
	skin+fat	100.1	——	12.0	——
	liver	97.7	——	2.6	——
	egg	99.0-104.1	101.0	2.5-3.3	3.5
fish	skin+muscle	88.9-105.9	95.0	3.6-4.6	6.4
shrimp	muscle	98.9-104.4	101.1	1.9-3.2	3.6

Table S10 Accuracy and precision of the method for rifaximin-High (180 ng/g)

animal	tissue	accuracy %		precision %	
		in-batch	between batch	in-batch	between batch
cattle	muscle	99.5-110.2	104.5	1.2-4.8	5.5
	fat	93.3-110.6	101.9	2.2-4.3	9.3
	liver	102.9-104.4	103.7	5.8-7.0	5.9
	kidney	99.8-106.8	103.3	2.7-5.9	5.0
	milk	93.6-113.6	101.3	4.0-5.3	10.4
swine	muscle	102.3	——	5.9	——
	skin+fat	96.8	——	6.7	——
	liver	97.3	——	6.8	——
	kidney	101.8	——	4.3	——
chicken	muscle	106.1	——	4.9	——
	skin+fat	105.9	——	5.7	——
	liver	100.4	——	3.5	——
	egg	100.0-103.9	102.1	3.4-3.9	3.8
fish	skin+muscle	88.8-105.9	98.6	2.0-7.3	9.0
shrimp	muscle	97.6-107.3	102.2	1.1-4.4	4.8

Table S11 Accuracy and precision of the method for rifampicin-LOD

animal	tissue	accuracy %		precision %	
		in-batch	between batch	in-batch	between batch
cattle	muscle	101.4-118.9	110.5	2.4-5.5	7.8
	fat	110.9-116.0	114.6	8.6-14.0	10.8
	liver	99.3-116.5	109.7	4.3-5.8	8.7
	kidney	95.3-116.4	106.0	2.0-3.6	8.9
	milk	91.9-126.9	109.7	5.7-8.5	15.0
swine	muscle	111.1	——	5.1	——
	skin+fat	115.6	——	3.4	——
	liver	114.3	——	3.8	——
	kidney	97.1	——	3.4	——
chicken	muscle	108.0	——	4.4	——
	skin+fat	113.5	——	3.4	——
	liver	98.3	——	1.9	——
	egg	109.0-113.3	1111	2.4-9.4	6.5
fish	skin+muscle	96.6-115.2	108.5	3.1-5.9	9.1
shrimp	muscle	98.8-112.0	106.7	1.4-5.7	6.4

Table S12 Accuracy and precision of the method for rifampicin-LOQ

animal	tissue	accuracy %		precision %	
		in-batch	between batch	in-batch	between batch
cattle	muscle	90.2-110.1	100.7	4.9-6.2	9.8
	fat	100.8-109.2	103.9	5.0-11.7	8.4
	liver	101.6-111.6	107.0	5.2-7.5	7.0
	kidney	98.7-102.9	101.5	2.1-5.7	4.2
	milk	105.7-115.9	110.2	4.1-10.4	7.9
swine	muscle	114.0	——	5.1	——
	skin+fat	110.1	——	3.4	——
	liver	104.6	——	3.2	——
	kidney	93.6	——	2.1	——
chicken	muscle	109.6	——	2.8	——
	skin+fat	108.0	——	3.8	——
	liver	100.4	——	1.9	——
	egg	104.6-107.8	106.7	3.3-5.2	4.0
fish	skin+muscle	92.6-113.8	101.5	4.2-5.6	9.8
shrimp	muscle	103.3-106.1	104.8	2.8-3.7	3.5

Table S13 Accuracy and precision of the method for rifampicin-2LOQ

animal	tissue	accuracy %		precision %	
		in-batch	between batch	in-batch	between batch
cattle	muscle	95.2-104.7	101.3	3.5-6.1	6.6
	fat	93.9-102.8	95.9	4.8-9.5	7.1
	liver	98.5-107.6	102.7	3.6-8.7	7.8
	kidney	98.3-109.1	105.5	1.4-4.6	5.7
	milk	101.1-111.3	107.5	4.3-5.3	6.1
swine	muscle	103.6	——	3.2	——
	skin+fat	105.9	——	6.7	——
	liver	96.0	——	3.8	——
	kidney	91.2	——	3.4	——
chicken	muscle	110.6	——	4.3	——
	skin+fat	97.3	——	4.2	——
	liver	98.5	——	4.1	——
	egg	93.2-106.34	98.9	4.5-10.1	9.0
fish	skin+muscle	95.5-106.9	99.9	3.1-9.1	8.1
shrimp	muscle	101.3-104.8	102.7	1.1-7.5	4.8

Table S14 Accuracy and precision of the method for rifampicin-Low (30 ng/g)

animal	tissue	accuracy %		precision %	
		in-batch	between batch	in-batch	between batch
cattle	muscle	99.6-106.6	103.5	2.0-5.3	4.5
	fat	93.9-102.7	98.3	3.9-8.4	6.1
	liver	95.7-102.6	99.7	2.8-5.6	5.1
	kidney	100.1-113.1	107.1	2.2-4.0	6.0
	milk	102.6-113.8	109.4	2.3-6.0	6.3
swine	muscle	106.0	——	1.9	——
	skin+fat	108.6	——	6.9	——
	liver	96.4	——	3.1	——
	kidney	92.5	——	3.1	——
chicken	muscle	102.0	——	9.0	——
	skin+fat	97.9	——	4.7	——
	liver	97.5	——	2.7	——
	egg	94.3	95.7	1.7-5.4	3.5
fish	skin+muscle	96.1-104.5	100.5	2.8-5.2	5.3
shrimp	muscle	98.0-101.2	99.7	3.8-7.2	5.3

Table S15 Accuracy and precision of the method for rifampicin-Medium (60 ng/g)

animal	tissue	accuracy %		precision %	
		in-batch	between batch	in-batch	between batch
cattle	muscle	101.3-103.8	102.9	1.9-4.2	3.5
	fat	97.0-97.7	97.6	2.3-10.0	6.1
	liver	98.2-105.9	101.3	3.9-5.6	5.8
	kidney	101.1-105.8	103.2	3.6-5.0	4.7
	milk	105.0-111.5	109.2	3.8-7.8	6.4
swine	muscle	103.4	——	4.0	——
	skin+fat	111.1	——	5.4	——
	liver	99.4	——	2.0	——
	kidney	93.5	——	4.7	——
chicken	muscle	105.7	——	4.7	——
	skin+fat	95.9	——	7.7	——
	liver	97.7	——	3.0	——
	egg	98.1-102.9	99.5	4.0-5.3	5.2
fish	skin+muscle	94.4-104.6	998	4.0-5.8	6.3
shrimp	muscle	99.1-102.9	102.2	3.1-6.6	5.2

Table S16 Accuracy and precision of the method for rifampicin-High (180 ng/g)

animal	tissue	accuracy %		precision %	
		in-batch	between batch	in-batch	between batch
cattle	muscle	99.6-105.9	103.1	1.9-3.1	3.6
	fat	90.9-105.5	98.2	6.9-10.9	10.1
	liver	95.7-102.9	99.4	3.5-8.7	6.6
	kidney	96.3-100.8	99.5	2.7-5.0	4.5
	milk	89.9-111.7	99.8	3.7-4.9	10.6
swine	muscle	100.4	——	3.7	——
	skin+fat	100.6	——	7.5	——
	liver	98.3	——	6.2	——
	kidney	100.6	——	4.2	——
chicken	muscle	97.8	——	3.9	——
	skin+fat	101.2	——	4.2	——
	liver	99.6-106.6	——	2.3	——
	egg	92.6-107.6	100.7	3.4-9.1	8.5
fish	skin+muscle	87.9-99.2	95.1	1.9-9.3	8.6
shrimp	muscle	97.4-104.1	100.3	4.3-7.1	6.1
